# Recent developments and future perspectives for photoresponsive-based gold nanoparticles (Au-NPs) and their biomaterial applications

**DOI:** 10.1039/d5ra06609c

**Published:** 2025-12-08

**Authors:** Mani Rajasekar, Meenamigai Sivakumar

**Affiliations:** a Centre for Molecular and Nanomedical Sciences, International Research Centre, Sathyabama Institute of Science and Technology (Deemed to be University) Chennai – 600 119 Tamil Nadu India mrajasekar_83@yahoo.com drmrajasekar.irc@sathyabama.ac.in; b Department of Biotechnology, School of Bioengineering, College of Engineering and Technology, SRM Institute of Science and Technology SRM Nagar, Chengalpattu Dist. Kattankulathur Tamil Nadu 603203 India

## Abstract

Photoresponsive gold nanoparticles (AuNPs) represent a dynamic class of nanomaterials combining the optical tunability of gold with the reversible photochemistry of organic chromophores. Functionalisation with azobenzene, fluorescein, perylene, and pyrene derivatives enables light-controlled modulation of molecular conformation, fluorescence, and surface interactions. These hybrid systems have shown wide applicability in drug delivery, imaging, biosensing, and photothermal therapy. This review summarises recent advances in their design, synthesis, and biomedical integration, with emphasis on photoisomerisation dynamics, optical coupling, and structure–function relationships. Key challenges including limited light penetration, photostability, and clinical scalability are highlighted alongside emerging prospects such as NIR-responsive and multi-stimuli platforms. Photoresponsive AuNPs thus offer a promising foundation for next-generation light-regulated biomaterials and precision nanomedicine.

## Abstract 2

Photoresponsive gold nanoparticles use light-active chromophores to enable reversible optical control, supporting advanced applications in imaging, sensing, and delivery, and offering potential for next-generation light-regulated biomaterials.

## Graphical abstract

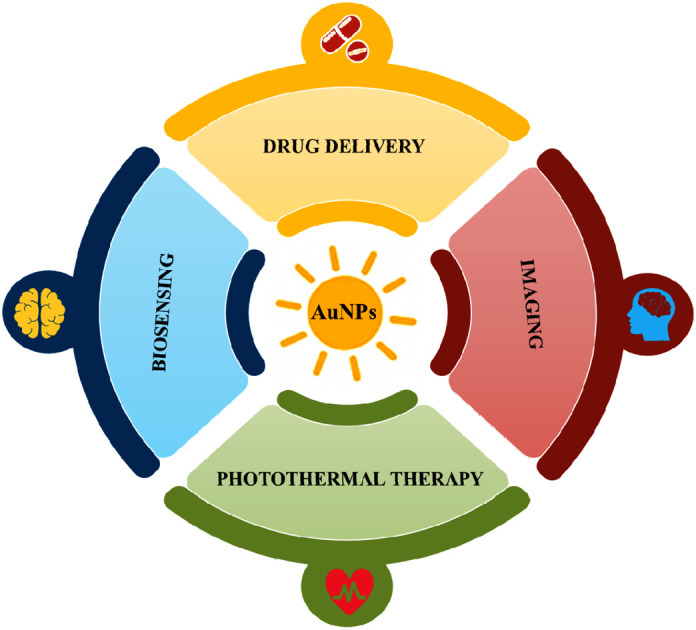

## 1. Introduction

Light-responsive nanomaterials have emerged as one of the most dynamic areas in nanoscience owing to their ability to reversibly modulate optical, chemical, and structural properties upon illumination.^1–5^ Among these, gold nanoparticles (AuNPs) occupy a central role due to their localized surface plasmon resonance (LSPR), high biocompatibility, and facile surface functionalization.^6^ The unique plasmonic behaviour of AuNPs allows efficient conversion of light into heat, tunable scattering and absorption, and strong coupling with photochromic molecules, thereby enabling precise spatiotemporal control of molecular and nanoscale events. The development of photoresponsive AuNPs can be broadly divided into three evolutionary stages. The first stage (early 2000s) focused on plasmonic photothermal therapy, where AuNPs were used as local heat transducers for cancer ablation. These pioneering studies established the concept of light-to-heat energy conversion and opened avenues for biomedical applications.^7^ The second stage (2010–2015) witnessed the introduction of photochromic organic ligands-such as azobenzene, spiropyran, and fluorescein onto AuNP surfaces, enabling reversible control of aggregation, surface polarity, and optical response through photoisomerization or fluorescence switching.^8^ The most recent stage (2015–2025) has expanded toward multifunctional and hybrid nanoplatforms, integrating photothermal, fluorescent, and optoelectronic functions for biosensing, imaging, and targeted therapy.^9^

The photoresponsive functional groups exhibit distinct photochemical mechanisms and application scopes when conjugated with AuNPs. Azobenzene derivatives undergo reversible *trans*–*cis* isomerization, providing efficient light-controlled switching of polarity and assembly.^10,11^ Fluorescein offers high fluorescence quantum yield and pH sensitivity, making it suitable for biosensing and bioimaging. Perylene derivatives exhibit strong π–π stacking and exceptional photostability, advantageous for optoelectronic and solid-state sensing applications. Pyrene derivatives display dual monomer–excimer emission, allowing ratiometric fluorescence sensing and environmental detection. Other photochromic moieties, such as spiropyran, which converts reversibly to its merocyanine form upon light irradiation and plasmon-induced polymerization systems where AuNP plasmons generate localized hot carriers to trigger polymerization, also represent important directions in the field of light-activated nanostructures.^12^ The literature surveyed in this review was selected to represent both the historical progression and application diversity of photoresponsive AuNPs. We prioritized studies that directly functionalised AuNPs or related nanostructures with photoactive chromophores. It demonstrated mechanistic or optical novelty and provided quantitative or reproducible data. They covered distinct application domains such as drug delivery, photothermal therapy, biosensing, imaging, and optoelectronics.^13–20^ Representative works were chosen to balance classical contributions with recent advancements, ensuring a comprehensive yet focused overview.

**Fig. 1:**
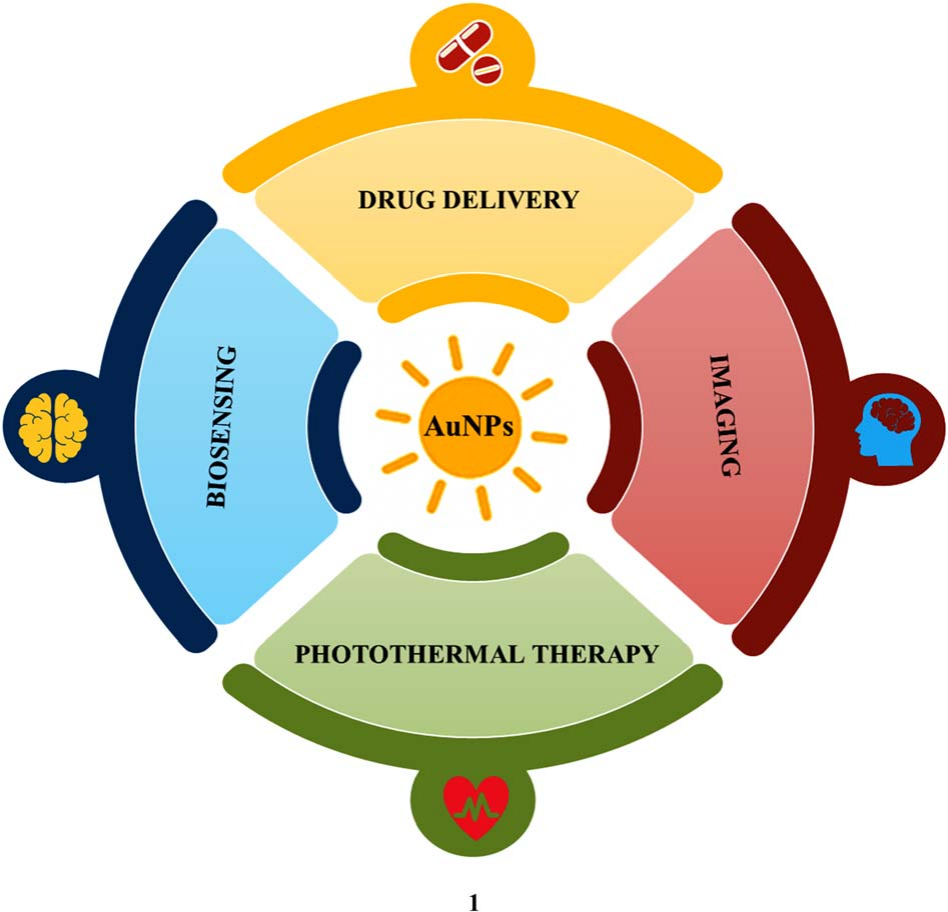
Schematic representation of photoactive gold nanoparticles (Au-NPs) and their biomedical applications (1).

This review is organized according to molecular type and functional mechanism, covering azobenzene, fluorescein, perylene, and pyrene-based AuNP systems (1). Each section traces the chronological evolution of research, highlights performance metrics, discusses advantages and limitations, and identifies emerging trends. A comparative summary and perspective are provided in the concluding section to aid researchers seeking to design the next generation of light-responsive AuNPs for biomedical and technological applications (Fig. 1).

## 2. Azobenzene-based gold nanoparticles

**Fig. 2:**
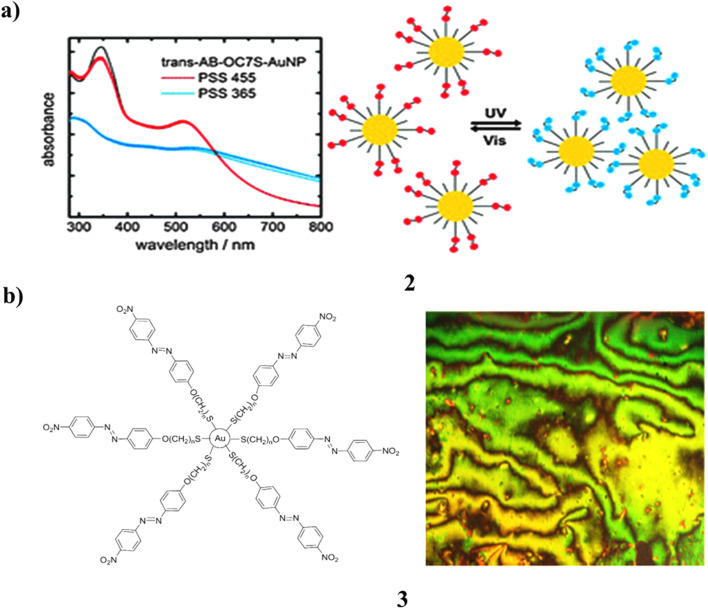
(a) Absorbance spectra showing photoisomerization of azobenzene-functionalized AuNPs (2) reproduced from ref. 21 with permission from Royal Society of Chemistry Publisher, Copyright 2014. (b) Reversible light-triggered *trans*–*cis* switching in azobenzene-thiol AuNPs (3) reproduced from ref. 22 with permission from Elsevier Publisher, Copyright 2016.

The photochemical properties of functionalized gold nanoparticles (AuNPs) featuring azobenzenethiolate–alkylthiolate monolayers (2) were systematically examined. It was demonstrated that repeated *trans*–*cis* and *cis*–*trans* isomerization cycles could be executed with remarkable efficiency across all studied cases. Notably, reversible photoinduced aggregation was observed when azothiolates possessing lengthy alkyl spacers (≥C_7_) were utilized in conjunction with shorter (C_5_) alkylthiolate coligands. Consequently, the selection of a coligand provides a means of modulation concerning the aggregation characteristics of the nanoparticles (Fig. 2a).^21^ A series of novel azobenzene-based thiolated liquid crystals (3) integrated with gold nanoparticles was synthesized and characterized employing various methodologies with delegated instrumentation. Investigations utilizing polarized optical microscopy revealed that all examined compounds exhibited liquid crystalline characteristics, specifically manifesting a typical nematic phase. The structural elucidation of these liquid crystal-capped gold nanoparticles was conducted *via* transmission electron microscopy (TEM) experiments. Moreover, azobenzene-based AuNPs featuring flexible spacers demonstrated photochromic behavior upon exposure to ultraviolet irradiation. The molecular entities showed pronounced photoisomerization behavior in solution with their *trans* to *cis* isomerization occurring within approximately 44 seconds, whereas the reverse process transpired over a time frame ranging from 82 to 125 minutes (Fig. 2b).^22^

**Fig. 3:**
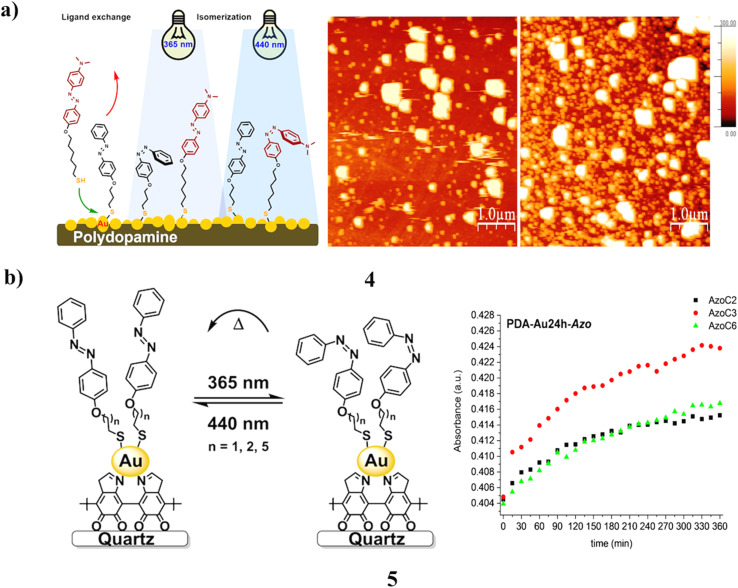
(a) AFM image of azobenzene-functionalized PDA-Au nanoparticles (4) reproduced from ref. 23 with permission from Wiley-VCH Publisher, Copyright 2020. (b) Thermal relaxation studies of azobenzene-PDA-based AuNPs (5) reproduced from ref. 24 with permission from Wiley-VCH Publisher, Copyright 2022.

The synthesis of dynamic interfaces was achieved by integrating photoisomerizable azobenzenes with polydopamine (PDA)/Au nanoparticle composites (4). Azobenzenes with different spacer lengths and surface anchoring groups were prepared. A polymeric layer was formed on quartz substrates through the simple aerobic autopolymerization of dopamine hydrochloride under alkaline conditions. The redox-active catechol groups within PDA enabled the *in situ* formation of gold nanoparticles on the polymer surface. UV-vis spectroscopic analyses confirmed that, upon successful assembly, the photo-switching behavior of azobenzenes on PDA/Au remained independent of both the spacer length and the anchoring moiety under the tested conditions. Moreover, due to the curved morphology of the Au nanoparticles, the surface-bound azobenzene layer could undergo reconstruction *via* ligand exchange, followed by photochemical characterization of the resulting mixed layer (Fig. 3a).^23^ Morphologically distinct aggregates of gold nanoparticles (5, AuNP) were prepared on macroscopic surfaces that were coated with a layer of polydopamine (PDA). The degree of particle aggregation and the distribution of particle sizes were controllable through the duration of Au(iii) reduction, with the reduction process being solely catalyzed by the redox-active polymer. Brief reduction times yielded smaller particles accompanied by diminished levels of aggregation, while extended reduction periods resulted in larger average particle diameters and increased aggregation. The fabricated surfaces were characterized using UV-vis, AFM, and SKPFM, and further employed as solvent-free condensed phases to study the photochemical and thermal isomerization of tethered azobenzenes with different spacer lengths. It exhibited rapid and reversible light-induced switching, while the thermal *cis*-to-*trans* isomerization proceeded faster for particle-bound azobenzenes than for their solution-phase counterparts (Fig. 3b).^24^

**Fig. 4:**
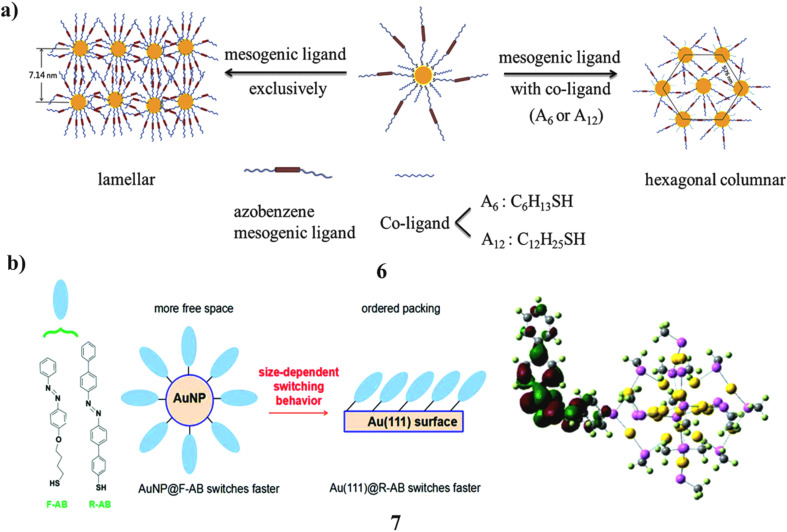
(a) Thermal response of azobenzene-mesogen-capped AuNPs showing reversible switching (6) reproduced from ref. 25 with permission from Elsevier Publisher, Copyright 2014. (b) LUMO analysis of azobenzene–Au(111) hybrid nanostructures (7) reproduced from ref. 26 with permission from Royal Society of Chemistry Publisher, Copyright 2017.

Alkoxyazobenzenemesogenic thiols featuring varying lengths of polyalkylene spacers have been synthesized and utilized as capping ligands, either exclusively or in conjunction with linear alkyl thiol co-ligands for the fabrication of hybrid gold nanoparticles (GNPs) (6). Subsequently, the thermal characteristics, phase behavior of the synthesized hybrid GNPs, and the photophysical properties of their solid-state films were examined employing the various spectroscopy techniques. In contrast to hybrid GNPs containing mixed ligands, which showed hexagonal columnar superstructures those that were only passivated with azobenzene mesogenic ligands showed a lamellar structure. Thermolysis resistance was markedly improved by the latter complex hybrid GNPs. Additionally, it is significant that the hybrid GNPs solid-state films demonstrated a reversible photoresponse, which is explained by the azobenzene mesogenic ligands *trans*–*cis* transformation. Compared to GNPs coated solely with mesogenic ligands, those functionalized with mixed ligands showed a faster photoisomerization rate under alternating UV and visible light, highlighting their potential for advanced photoresponsive molecular sensing applications (Fig. 4a).^25^ Biosensors and thermotherapy are only two of the potential uses for the photo-responsive azobenzene (7, AB) in combination with biocompatible gold nanoparticles. Two different azobenzene derivatives and three different gold substrates were taken to produce six different Au@AB arrangements in order to examine the impact of these components on the collective switching behavior. The collective photo-induced *cis*-to-*trans* switching process of AB monolayers on gold substrates was simulated using a RMD model that takes into consideration both torsion and inversion routes. The torsion of the C–N=N–C dihedral angle is found to be the main driving factor underlying isomerization with the inversion route contributing less. Slow self-organization is seen over 40 picoseconds during the relaxation step of the isomerization process, which is separated from the initial conformation change stage. The packing structure of the AB monolayer is influenced by the gold substrate, and the intermolecular interactions within the monolayer are modulated by the assortment of AB types. When compared to the surface, flexible alkoxyl-linked F-AB may convert on gold clusters much more quickly. The torsional movement of the C–N=N–C dihedral and the ensuing isomerization process may be hindered in the case of stiff biphenyl-based R-AB anchored to AuNPs by a competing torsional contact between the biphenyl and the dihedral. The strong π–π stacking between biphenyl units promotes the collective isomerization process when the R-AB molecules are fixed onto the Au(111) surface. The impact of RAB self-assembled monolayers (SAMs) on substrates of different diameters is seen to be curvature-dependent. The collective switching behavior of Au@AB materials is determined by the interaction between the gold substrate and functional AB monolayers. It is expected that these results will guide the logical development of Au@AB hybrid materials for a range of applications (Fig. 4b).^26^

**Fig. 5:**
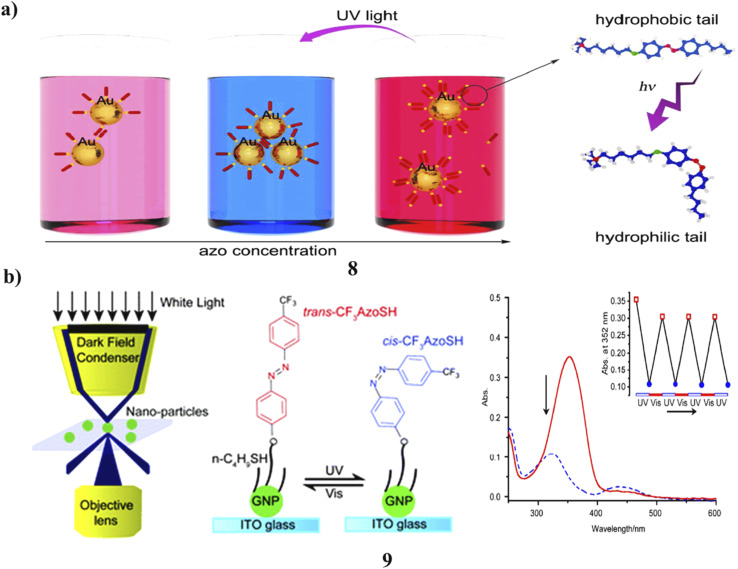
(a) UV-vis spectra of light-tunable plasmonic azobenzene-surfactant-AuNP complexes (8) reproduced from ref. 27 with permission from American Chemical Society Publisher, Copyright 2015. (b) Reversible photoisomerization of azobenzene-coated AuNPs (9) reproduced from ref. 28 with permission from Royal Society of Chemistry Publisher, Copyright 2016.

The plasmonic nanoarchitectures constructed from gold nanoparticle-azobenzene modified cationic surfactant complexes (8) demonstrate the capacity for light-tunable plasmonic responses. The generation of such complexes is facilitated by the utilization of bare gold nanoparticles, which are strongly negatively charged and synthesized through laser ablation techniques in deionized water. The electrostatic interactions facilitate the attachment of cationic surfactants, leading to the formation of a shell around the negatively charged nanoparticles. This process results in charge neutralization or even overcompensation, resulting in the nanoparticles acquiring a positive charge. At both low and high surfactant concentrations, the Au nanoparticles exhibit negative and positive charges, respectively, and are represented as individual entities due to electric repulsion, displaying absorption peaks in the range of 523 nm to 527 nm. In contrast, the Au nanoparticles achieve neutrality at intermediate surfactant concentrations, forming nano-sized aggregates about 100 nm in diameter. This effect is marked by an additional absorption peak at *λ* > 600 nm and a distinct color change of the solution from red to blue. The photosensitive azobenzene unit in the surfactant tail undergoes *trans*-to-*cis* isomerization under UV light, driving light-controlled nanoparticle clustering that shifts the plasmonic absorption band and alters the solution's color. The resultant hybrid architectures with light-responsive plasmonic characteristics hold significant potential for various applications, including biomedical, SERS, data transmission, and storage (Fig. 5a).^27^ The photoresponsive behavior of CF_3_AzoSH molecules (9) on individual GNP surfaces was examined, revealing that their reversible photoisomerization directly corresponds to reversible shifts in single-particle spectra. The experimental results align well with theoretical predictions from DDA simulations. Notably, the rate of red shift in scattered spectra provides valuable insight into the photoisomerization kinetics of CF_3_AzoSH on single GNPs and the associated surface-induced quenching effects. Importantly, this approach is not limited to CF_3_AzoSH but can be broadly applied to study the photoisomerization of other azobenzene derivatives on individual GNP surfaces. The outcomes of this research indicate that PRRS spectroscopy holds considerable promise for examining molecular behavior at the level of single nanoparticles. Moreover, the exploration of photoisomerization processes at the single nanoparticle level bears substantial significance for the advancement of nano-sized “write-read” devices (Fig. 5b).^28^

**Fig. 6:**
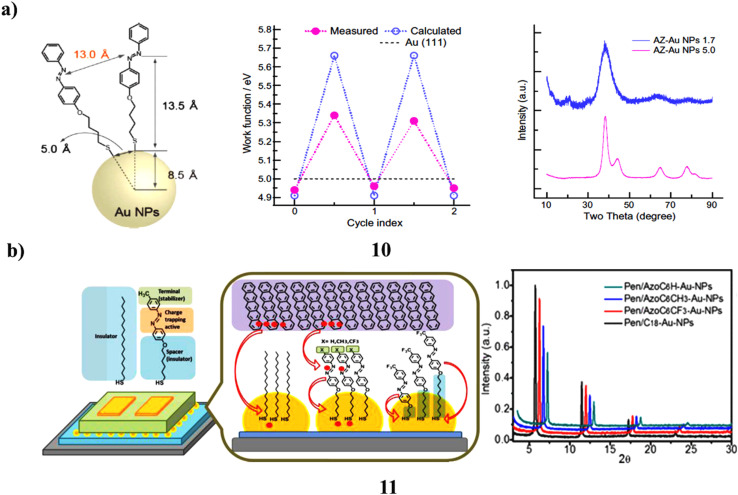
(a) Photoinduced magnetic modulation in azobenzene-thiol-gold nanoparticles (10) reproduced from ref. 29 with permission from Elsevier Publisher, Copyright 2009. (b) XRD pattern of pentacene films deposited on azobenzene-AuNP interfaces (11) reproduced from ref. 30 with permission from American Chemical Society Publisher, Copyright 2013.

The synthesized gold nanoparticles passivated by azobenzene-thiol (10) exhibit a range of sizes. Observations within these systems indicate that a reduction in size to 1.7 nm fosters the emergence of ferromagnetism even at ambient temperature, whereas a particle size of 5.0 nm is predominantly characterized by diamagnetism. Notably, the ferromagnetic properties can be modulated through alternating photonic stimulation with ultraviolet and visible light in a solid-state environment, maintaining effectiveness at room temperature. The variations in magnetization values were pronounced, approximated at 27%. This photo-magnetic phenomenon is postulated to stem from photo-induced alterations in d-charge loss values attributed to the photoisomerization of azo-ligands, which are concomitant with an inversion of surface dipole values to the contrary orientation (Fig. 6a).^29^ A self-assembled monolayer of azobenzene derivatives (11) encasing Au-NPs was created at the interface between a pentacene thin film and the dielectric insulator SiO_2_. These composite channel materials were used to fabricate transistors that showed electric bistability under different gate biases. The monolayer concurrently served as a barrier layer, a modulator of work function, and extra locations for charge trapping at the Au-NPs/semiconductor interface. The methyl substituted azobenzene-modified Au-NPs produced a transistor memory device with almost 70% more charges maintained a noticeably faster response time, and an improved retention lifetime compared to plain alkanethiol monolayer-covered Au-NPs. Additionally, the substituent on the azobenzene moieties and the length of the tethering alkyl chain may be used to adjust the rate of carrier trapping and the stability of the trapped carriers, improving device performance. The subsequent sections will elaborate on the structural and electronic attributes of these devices (Fig. 6b).^30^

**Fig. 7:**
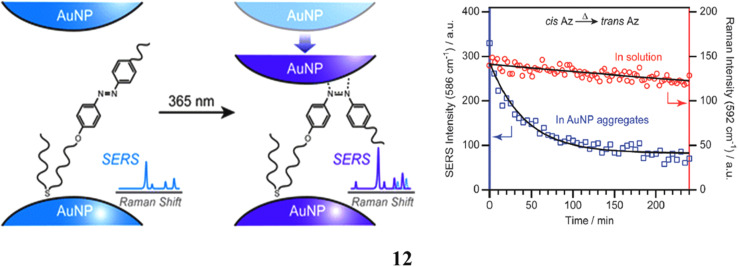
SERS spectra showing photoisomerization of azobenzene derivatives confined in AuNP aggregates (12) reproduced from ref. 31 with permission from Royal Society of Chemistry Publisher, Copyright 2011.

Photoisomerization represents a critical reaction that imparts photoresponsive properties to nanoparticles. While the photoisomerization of molecules constituting self-assembled monolayers on two-dimensional surfaces or three-dimensional clusters has been the subject of investigation, a comprehensive understanding of the interactions between isomerizing molecules and nanoparticles remains elusive. The photoisomerization of azobenzene derivatives (12), when spatially confined within AuNP aggregates is of particular interest. AuNP aggregates enable simultaneous monitoring of molecular structural changes *via* SERS and alterations in interparticle interactions through surface plasmon coupling. These aggregates are formed by adsorbing azobenzene-derivatized sulfides (Az) onto AuNP surfaces. Upon 365 nm irradiation, Az undergoes *trans*-to-*cis* isomerization, driving closer aggregation of AuNPs, which produces a redshift in the plasmon coupling band and an increase in SERS intensity. The SERS spectra further reveal that –N=N– stretching vibrations shift to lower frequencies upon irradiation, indicating bond weakening in the cis form. This effect arises from the greater exposure of the –N=N– bond in the cis configuration, which interacts more strongly with adjacent AuNPs brought closer together by Az isomerization. They discover that due to the lower NQN bond order of *cis*-Az in the aggregates, back isomerization from cis to trans happens far more quickly in the AuNP aggregates than in solution (Fig. 7).^31^

Azobenzene-based gold nanoparticles (Au-NPs) represent a promising class of light-responsive nanomaterials that leverage the reversible *trans*–*cis* isomerization of azobenzene under specific wavelengths of light. This photoisomerization alters the molecular geometry and polarity of azobenzene, leading to significant changes in the surface properties, assembly behavior, and functional activity of the gold nanoparticles. In terms of performance, these systems offer dynamic control over particle aggregation, surface hydrophobicity, and ligand accessibility, making them ideal for applications in targeted drug delivery, optical switching, and biosensing. The detection mechanism often relies on plasmonic shifts in UV-vis spectra or fluorescence quenching/enhancement triggered by light-induced conformational changes. Azobenzene-functionalized Au-NPs exhibit high selectivity when designed with complementary recognition units, enabling precise molecular sensing or controlled release in response to light. Advantages include their reversible and non-invasive activation, high responsiveness, and ease of functionalization. However, disadvantages include potential photofatigue (loss of switching efficiency over time), limited light penetration in biological tissues (especially with UV), and the need for careful molecular design to ensure biocompatibility and stability in physiological environments. Despite these challenges, azobenzene-based Au-NPs remain a versatile platform for developing smart, light-triggered biomaterials. Overall, azobenzene-functionalized AuNPs exhibit fast and reversible *trans*–*cis* photoisomerization with remarkable structural control, enabling light-regulated aggregation, wettability, and molecular recognition. Their distinct advantage lies in the rapid switching response (typically within 40–120 s) and robust reversibility under alternate UV-visible irradiation, making them particularly attractive for photothermal control and dynamic optical devices. However, the requirement for UV activation limits their penetration depth in biological systems, and repetitive cycles may induce photofatigue or ligand degradation. Compared to other photoresponsive systems, azobenzene-based AuNPs are best suited for nanoscale actuators, light-driven release systems, and optoelectronic switching platforms where localized surface control is desirable.

## 3. Fluorescein-based gold nanoparticles

**Fig. 8:**
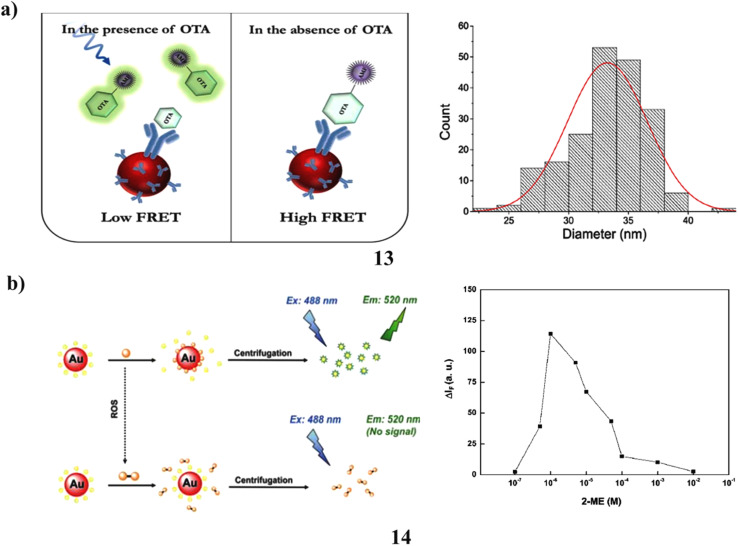
(a) FRET-based fluorescence sensing of ochratoxin A using fluorescein-AuNP conjugates (13) reproduced from ref. 32 with permission from Elsevier Publisher, Copyright 2022. (b) Fluorescent detection of reactive oxygen species by fluorescein isothiocyanate-AuNPs (14) reproduced from ref. 33 with permission from Royal Society of Chemistry Publisher, Copyright 2010.

The identification of ochratoxin A (OTA), a detrimental toxic contaminant present in food products that can lead to severe health issues, is of paramount significance. Conventional analytical techniques employed for this purpose are often costly and require substantial time and labor investment. A FRET-based immunosensor (13) was developed for detecting ochratoxin A (OTA). In this system, a fluorophore acts as the energy donor, while AuNPs serve as the energy acceptor. This design enables sensitive and specific OTA detection. Due to the competitive immunoreaction between free OTA and OTA-AMF for binding to anti-OTA antibodies coupled with AuNPs the fundamental sensing concept depends on detecting the recovered fluorescence of OTA-AMF. A linear connection between the OTA-AMF fluorescence intensity and the OTA concentration was found under ideal experimental circumstances. This association has a detection limit of 0.02 ng mL^−1^ and varied from 0.09 to 3.12 ng mL^−1^. The proposed fluorescent immunosensor was validated using wheat and wine samples, achieving recovery rates of 70.8–115.8% with relative standard deviations below 10%. This work highlights a rapid and user-friendly sensing platform based on the antigen-antibody-mediated FRET mechanism of the AMF-AuNP donor–acceptor pair (Fig. 8a).^32^ Additionally, the oxidation of 2-mercaptoethanol (2-ME) and the resulting increase in fluorescence of fluorescein isothiocyanate-capped gold nanoparticles (14, FITC-AuNPs) establish a simple and highly sensitive technique for the fluorescent detection of reactive oxygen species (ROS). It is easy to conjugate FITC molecules to the surface of citrate-capped AuNPs through their isothiocyanate group. The effective energy transfer from FITC to AuNPs is the main cause of the weak fluorescence seen in FITC-AuNPs. FITC's fluorescence has been reported to be revived when 2-ME is present because it promotes the separation of FITC from the AuNP surface by developing Au–S bonds. FITC cannot be removed off the surface of the nanoparticle by the 2-ME disulfide that results from the oxidation of 2-ME by ROS in an alkaline environment. The addition of increasing ROS concentrations therefore caused FITC-AuNPs' feeble fluorescence to gradually rise. This method's sensitivity to ROS was much increased when the AuNPs were removed by centrifugation. This technique was also used for glucose detection since the oxidation of glucose, which is mediated by glucose oxidase results in the production of gluconic acid and H_2_O_2_. Under optimal conditions, the detection limits for H_2_O_2_, superoxide anion, hydroxyl radical, and glucose were 1, 0.6, 0.6, and 1 µM, respectively. This approach was further validated as an effective method for quantifying glucose in blood samples (Fig. 8b).^33^

**Fig. 9:**
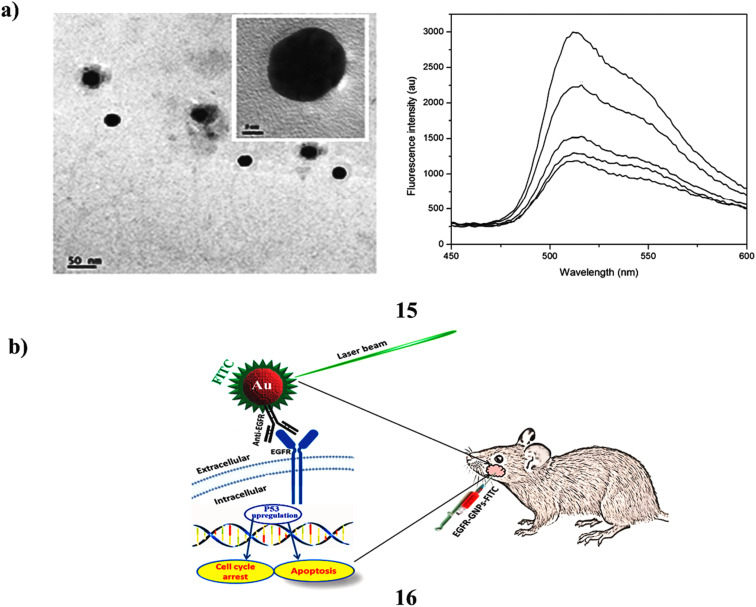
(a) Optical emission characteristics of green-synthesized fluorescein-AuNP systems (15) reproduced from ref. 34 with permission from Royal Society of Chemistry Publisher, Copyright 2015. (b) CLSM imaging of tumour cells targeted with Anti-EGFR-fluorescein-AuNP conjugates (16) reproduced from ref. 35 with permission from Elsevier Publisher, Copyright 2022.

The capacity of dye-nanoparticle composites to alter the emission properties of dye by varying nanoparticle parameters makes them very promising for use in biological and photonics applications. However, current research does not provide a thorough knowledge of homogenous dye and nanoparticle combinations, in contrast to the well-specified distance between dye and nanoparticles. The optical characteristics of fluorescein dye in a nanoparticle mixture are investigated in this work in relation to the concentration and form of gold nanoparticles (15) produced using eco-friendly techniques. The non-radiative mechanism of de-excitation was examined using a laser-based dual-beam thermal lens approach, while the radiative pathway was examined using steady-state fluorescence. The efficiency of energy transfer and the distance between dye and nanoparticles were assessed through both methodologies. The researchers also used the thermal lens approach to investigate the properties of nanoparticles affected the fluorescence quantum yield of fluorescein. The results show that star-shaped nanoparticles may investigate further distances between dye and nanoparticles, whereas spherical nanoparticles are efficient quenchers. The modification of dye characteristics through the adjustment of nanoparticle parameters holds potential applications across various fields, including bioimaging, solar cells, and sensors (Fig. 9a).^34^ The genesis and spread of cancer are significantly influenced by the epidermal growth factor receptor (EGFR). When paired with antibodies, gold nanoparticles (GNPs) show remarkable effectiveness in detecting and identifying malignancies. This study used a confocal laser scanning microscope (CLSM) to target tumor-induced cells in hamster mucosal tissue by conjugating GNPs with Anti-EGFR and then labeling them with FITC. Anti-EGFR-GNPs-FITC (16) was synthesized by mixing 10 mL of gold solution with 1 mL of antibody solution. To stabilize the conjugate and prevent aggregation, 0.5 mL of 1% PEG was added. The mixture was mixed with FITC and swirled all night. Transmission electron microscopy, and zeta potential analysis were used to characterize GNPs and Anti-EGFR-GNPs-FITC. The CLSM examination of tissue treated with Anti-EGFR-GNPs-FITC demonstrates effective distribution and bioconjugation of Anti-EGFR-GNPs-FITC within tumor cells. The optimal timeframe for distribution within tumor tissue was identified as between 6 and 8 hours. Furthermore, a real-time PCR analysis indicated an increase in the average expression levels of the P53 tumor suppressor gene in both normal and tumor-induced cells. The administration of anti-EGFR-GNPs-FITC may help to suppress the growth of tumour cells as shown by the up-regulation of P53 gene expression. The new GNP-antibody assembly (Anti-EGFR-GNPs-FITC) described in this study effectively accomplished bioconjugation and tumour suppression in mucosal cells (Fig. 9b).^35^

**Fig. 10:**
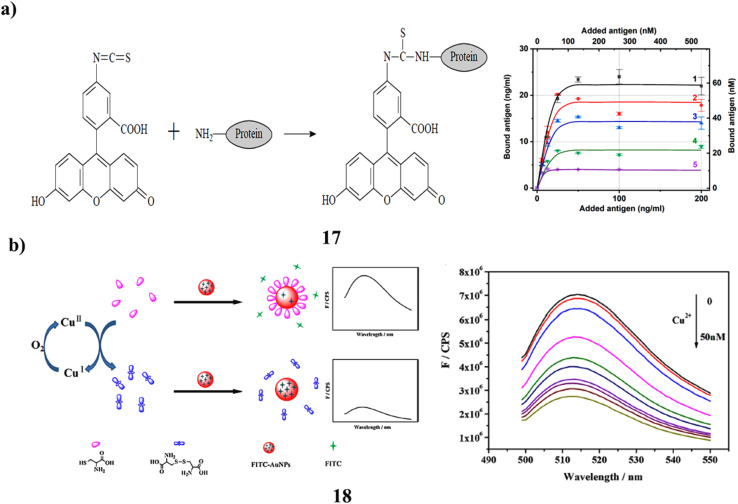
(a) Antibody–antigen binding efficiency on aminofluorescein-gold nanoparticles (17) reproduced from ref. 36 with permission from MDPI Publisher, Copyright 2023. (b) Fluorescence recovery in FITC-AuNPs for Cu^2+^ ion sensing (18) reproduced from ref. 37 with permission from Elsevier Publisher, Copyright 2015.

Antibodies are commonly used in analytical applications, often requiring their immobilization on various carriers. However, this process frequently results in a partial loss of their ability to bind antigens. While the behavior of antibodies on plain carriers is well-documented, there is limited information regarding their properties on nanoparticle surfaces. Most previous studies have focused on protein antigens, where spatial constraints prevent them from accessing all antibody binding sites. This study explores the interaction between antibodies and a small molecule antigen, fluorescein, using spherical gold nanoparticles of five different sizes. The researchers tested two forms of fluorescein with different charges, along with three levels of antibody surface coverage. For nanoparticles ranging in size from 14 to 35.5 nm and covered with a monolayer of antibodies, only 6% to 17% of the immobilized antibodies were capable of binding carboxyfluorescein. In contrast, the binding efficiency for aminofluorescein (17) was higher, reaching 27% on 21 nm nanoparticles, compared to 13% for carboxyfluorescein. Reducing the antibody coverage to one-fourth of a monolayer did not significantly affect the proportion of active binding sites. These results suggest that antigen binding is restricted even for small molecules, and is influenced by nanoparticle size and surface electrostatic interactions (Fig. 10a).^36^ Additionally, a novel fluorescence-based method was developed to sensitively detect copper ions (Cu^2+^) using gold nanoparticles functionalized with fluorescein isothiocyanate (18, FITC-AuNPs). The strong affinity between isothiocyanate groups and gold surfaces enables FITC to adsorb onto the nanoparticles developing a FRET system that quenches the FITC signal. When cysteine is introduced, it displaces FITC from the gold surface due to the higher formation constant of the Au–S bond compared to Au–SCN, thereby restoring fluorescence. The fluorescence recovery is low when Cu^2+^ is present because oxygen oxidises cysteine to cystine, which cannot displace FITC. This mechanism allows for the quantitative detection of Cu^2+^, with a detection range of 1.0–17.0 nM and a detection limit of 0.37 nM (3*σ*/*S*). The method is notable for its simplicity, high selectivity for Cu^2+^, and avoidance of complex synthesis or solubility issues (Fig. 10b).^37^

**Fig. 11:**
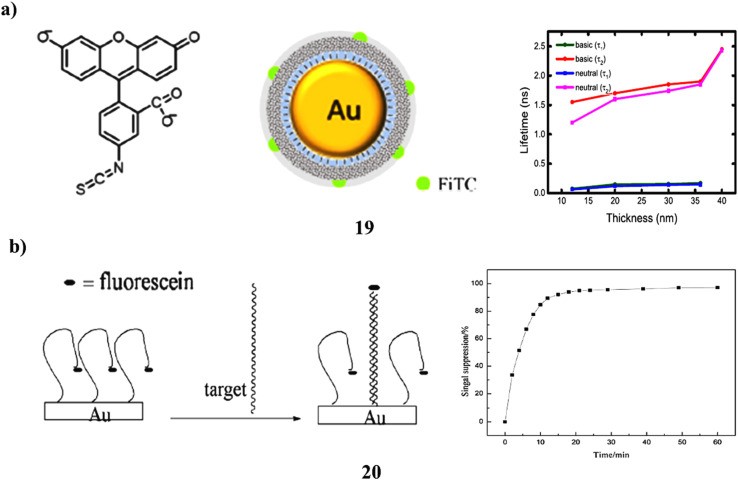
(a) Fluorescence enhancement in GNP@silica@FITC core–shell systems with varied shell thickness (19) reproduced from ref. 38 with permission from Elsevier Publisher, Copyright 2021. (b) Electrochemical DNA biosensor using fluorescein-labeled AuNP electrodes (20) reproduced from ref. 39 with permission from Elsevier Publisher, Copyright 2013.

Fluorescein-labeled core–shell NPs containing of a gold core-silica shell (GNP@silica@FITC (19)) were synthesized to investigate the effects of metal-enhanced fluorescence (MEF) in both neutral and basic ethanol solutions. Time-resolved fluorescence spectroscopy was used to analyze how the presence of gold nanoparticles influences the fluorescence of fluorescein with the silica shell acting as a spacer of varying thickness. Fluorescein's emission spectra were found to overlap with the surface plasmon resonance of gold nanoparticles, making this system suitable for studying MEF-related fluorescence enhancement. The emission from GNP@silica@FITC showed a red shift compared to both silica@FITC and fluorescein in solution. The fluorescence decay curves exhibited a biexponential pattern, suggesting multiple relaxation pathways for the excited-state fluorophores. Fluorescence enhancement factors (EFs) were slightly higher in neutral ethanol than in basic ethanol. The greatest enhancement (EF ≈ 2) occurred at the smallest silica shell thickness of approximately 12 nm, with enhancement decreasing as the shell thickness increased. At this 12 nm spacing, the short lifetime component of the emission decay was around 60–70 picoseconds and accounted for roughly 90% of the total amplitude. From the fluorescence lifetimes, amplitude ratios, and a proposed kinetic model, energy transfer rate constants between the fluorophores and gold nanoparticles were calculated, showing a clear dependence on the thickness of the silica shell (Fig. 11a).^38^ In a separate study, a novel electrochemical DNA biosensor (20) was developed using a gold electrode modified with gold nanoparticles, referred to as a nanogold electrode. This platform enabled both the immobilization of DNA probe strands and the adsorption of fluorescein onto the nanoparticle surfaces, forming an “arch-like” configuration. The sensor's performance was compared to that of linear DNA immobilized on an unmodified gold electrode. Results indicated that the nanogold modification improved sensitivity by enhancing fluorescein adsorption. The biosensor demonstrated a linear detection range for target single-stranded DNA (ssDNA) from 2.0 × 10^−9^ M to 2.0 × 10^−8^ M, with a high correlation coefficient (*R*^2^ = 0.9956) and a detection limit of 7.10 × 10^−10^ M (based on 3*σ*). The study also evaluated the sensor's specificity and its response to DNA hybridization (Fig. 11b).^39^

**Fig. 12:**
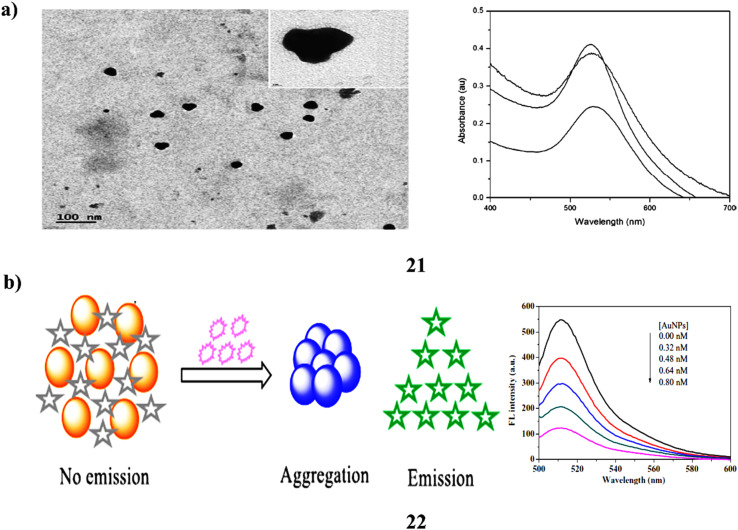
(a) Photophysical response of green-synthesized fluorescein-AuNP nanofluids (21) reproduced from ref. 40 with permission from Elsevier Publisher, Copyright 2016. (b) Fluorescent sensing of biothiols using fluorescein-AuNP probes (22) reproduced from ref. 41 with permission from Elsevier Publisher, Copyright 2020.

The interaction between dye molecules and gold nanoparticles (21) of different shapes has attracted significant attention due to its potential applications in bioimaging, sensing, photodynamic therapy, and photonics. Noble metal nanoparticles, particularly gold, are often used to either enhance or suppress the fluorescence of nearby dye molecules. However, most studies to date have worked with low dye concentrations to avoid self-quenching effects. This study goes further by exploring how gold nanoparticles synthesized using eco-friendly (green) methods affect the emission behavior and fluorescence quantum yield of fluorescein dye, even at concentrations where self-quenching typically occurs. The emission behavior was investigated using laser-based steady-state fluorescence, while quantum yield was measured with a dual-beam laser-based thermal lens technique. Researchers observed a reduction in fluorescence intensity alongside an increase in thermal lens signal near the nanoparticles. This shows that the dye (donor) and the nanoparticles (acceptor) are transferring energy non-radiatively. Additionally, the study examined how the pH of the gold nanofluid affected absorption, emission, and quantum yield. These findings reveal that even at high dye concentrations, both the shape and presence of gold nanoparticles significantly influence the dye's optical properties, an insight that could be valuable for future applications in fields like diagnostics and nanophotonics (Fig. 12a).^40^ A simple and effective “turn-on” fluorescence sensing method was developed to detect biothiols such as cysteine (Cys), homocysteine (HCys), and glutathione (GSH). These sulfur-containing amino acids can bind to gold nanoparticles *via* their thiol groups, causing the particles to aggregate. In their aggregated form, the gold nanoparticles can quench the fluorescence of fluorescein (22) due to an inner filter effect. However, when biothiols are introduced, they bind to the AuNPs and prevent further quenching, resulting in the restoration of fluorescein fluorescence. This fluorescence recovery was optimized using cysteine as a model compound by adjusting parameters such as AuNP concentration, pH, and incubation time at room temperature. Under optimal conditions, this method could sensitively detect Cys, HCys, and GSH with LOD for 0.027 µM, 0.023 µM, and 0.030 µM, respectively. Moreover, the sensor performed reliably when tested with human serum samples showing high precision and accuracy. Overall, the approach is promising due to its simplicity, sensitivity, and suitability for real-world biomedical applications (Fig. 12b).^41^

**Fig. 13:**
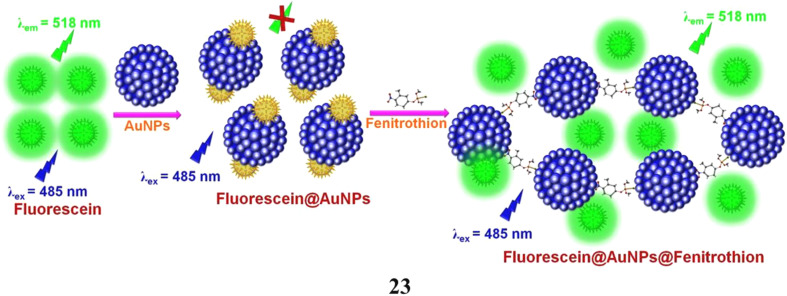
Fluorescence turn-on sensing of pesticide fenitrothion using fluorescein-AuNP probes (23) reproduced from ref. 42 with permission from Elsevier Publisher, Copyright 2018.

The NSET mechanism of AuNPs on the fluorescence of fluorescein has been used to develop a simple and sensitive technique for determining fenitrothion (23). The fluorescein system quenched by gold nanoparticles has remarkable sensitivity and selectivity towards fenitrothion, even when a variety of pesticides, anions, and cations are present. The gold nanoparticle-quenched fluorescein probe's fluorescence turn-on response to fenitrothion has been effectively shown on a paper strip. Analysis of real water samples from various sources produced detection limits of 6.05 nM for well water, 9.41 nM for tap water, and 7.84 nM for river water. The fluorescein fluorescence was reversibly quenched by the Fe^3+^ ions in iron oxide nanoparticles. It was unable to separate and sense fenitrothion at the same time because this inhibited the turn-on response. They also used superparamagnetic iron oxide to magnetically recover fenitrothion linked to AuNPs from the contaminated water (Fig. 13).^42^

Fluorescein-based gold nanoparticles (Au-NPs) combine the strong fluorescence properties of fluorescein dyes with the unique optical and surface characteristics of AuNPs resulting in multifunctional nanoplatforms for bioimaging and sensing. The principle relies on the interaction between the fluorescein molecules and the Au-NP surface, where fluorescence can be modulated through mechanisms such as FRET depending on the proximity and orientation of the dye to the gold core. In terms of performance, these nanoconjugates exhibit high sensitivity and brightness, making them suitable for real-time tracking, cellular imaging, and trace-level analyte detection. The detection mechanism typically involves light-induced fluorescence emission, where changes in intensity or wavelength signal the presence of target molecules or environmental changes. These systems demonstrate good selectivity when fluorescein is chemically modified to respond to specific targets or conjugated with selective recognition elements such as antibodies or aptamers. Advantages include high fluorescence quantum yield, biocompatibility, ease of conjugation, and suitability for optical diagnostics. However, disadvantages involve potential photobleaching of fluorescein, pH sensitivity affecting fluorescence stability, and possible quenching effects at high nanoparticle loading or aggregation. Despite these limitations, fluorescein-based Au-NPs are generally used in bio-imaging, and biosensing due to their strong optical responsiveness. In summary, fluorescein-AuNP conjugates combine strong optical brightness with tunable fluorescence modulation through FRET and surface plasmon interactions. Their high quantum yield (≈0.9) and low detection limits (as low as 0.02 ng mL^−1^ for OTA and 0.37 nM for Cu^2+^) underscore their superior analytical sensitivity. Unlike azobenzene systems, these conjugates rely on emission-based signaling rather than conformational change, rendering them ideal for real-time biosensing and imaging. Their performance, however, is influenced by pH-dependent emission stability and photobleaching upon prolonged excitation. Hence, fluorescein-modified AuNPs are optimally employed in fluorescence imaging, point-of-care diagnostics, and environmental monitoring where high signal intensity is critical.

## 4. Perylene-based gold nanoparticles

**Fig. 14:**
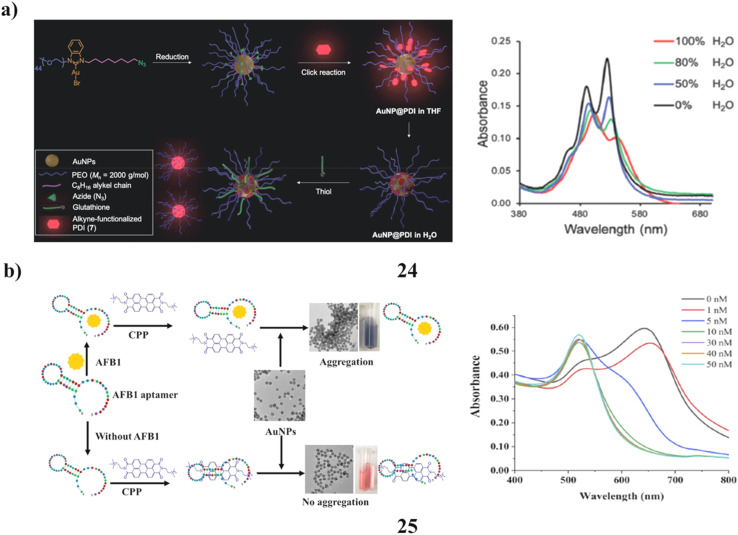
(a) Absorbance spectra of PDI-NHC-AuNP conjugates (24) reproduced from ref. 43 with permission from Royal Society of Chemistry Publisher, Copyright 2021. (b) Photophysical analysis of aflatoxin B_1_-perylene-AuNP aptasensor (25) reproduced from ref. 44 with permission from Elsevier Publisher, Copyright 2020.

N-heterocyclic carbenes (NHCs) have recently gained attention as effective alternatives to traditional thiols for stabilizing metal surfaces and nanoparticles. In particular, NHC-stabilized gold nanoparticles (AuNPs) have been widely studied owing to their biocompatibility and distinctive optical properties. These systems exhibit remarkable stability under acidic and basic conditions, high temperatures, electrolyte solutions, cell culture media, and even to some extent in the presence of nucleophilic thiols. Despite rigorous research, the instability of NHC-functionalized AuNPs when exposed to thiols remains a persistent challenge. To address this issue, the quantification of NHC desorption from the nanoparticle surface induced by the encroaching thiols is a critical initial step. The first iteration of water-soluble azide-decorated NHC-stabilized “clickable” AuNPs has been developed. Optically active perylenediimide (PDI)-tagged AuNP hybrids (24) were synthesized *via* Cu-catalyzed alkyne–azide cycloaddition between AuNPs and an alkyne-functionalized PDI derivative. Photophysical studies revealed strong fluorescence quenching of PDI by AuNPs in aqueous media. The stability of NHC ligands on AuNPs was evaluated in the presence of glutathione (4 mM), where fluorescence recovery of detached PDI indicated ligand displacement. Results showed that \∼20% of NHCs were displaced within 24 hours, increasing to ∼45% after one week. These findings provide valuable insights into the actual stability of NHC-stabilized AuNP systems (Fig. 14a).^43^ A label-free colorimetric aptasensor (25) has been developed for the rapid and straightforward detection of aflatoxin B_1_ (AFB_1_), utilizing a G-rich specific aptamer (CPP) and unmodified citrate-stabilized gold nanoparticles (AuNPs). The sensing mechanism is based on the localized surface plasmon resonance (LSPR) absorption properties of AuNPs, which change upon aptamer–toxin interaction. Critical parameters influencing the performance of the sensor include CPP concentration, aptamer quantity, and the pH of the medium. The developed method exhibited linear detection ranges of 1–10 ng mL^−1^ when measured using a spectrophotometer and 1–6 ng mL^−1^ with a custom-built LED-LDR colorimeter. The aptasensor demonstrates key advantages such as simplicity, selectivity, and rapid response, making it highly practical for routine analysis. Notably, the LED-LDR colorimeter offers a portable and cost-effective alternative to traditional spectrophotometers, without compromising sensitivity, and is well suited for on-site monitoring applications. Both detection platforms achieved sensitivities appropriate for real-world testing scenarios. To further validate its applicability, the aptasensor was integrated with an affinity column for sample preparation and successfully applied to detect AFB1 in real samples, delivering reliable accuracy and precision. These results highlight its potential as a practical and efficient tool for mycotoxin monitoring (Fig. 14b).^44^

**Fig. 15:**
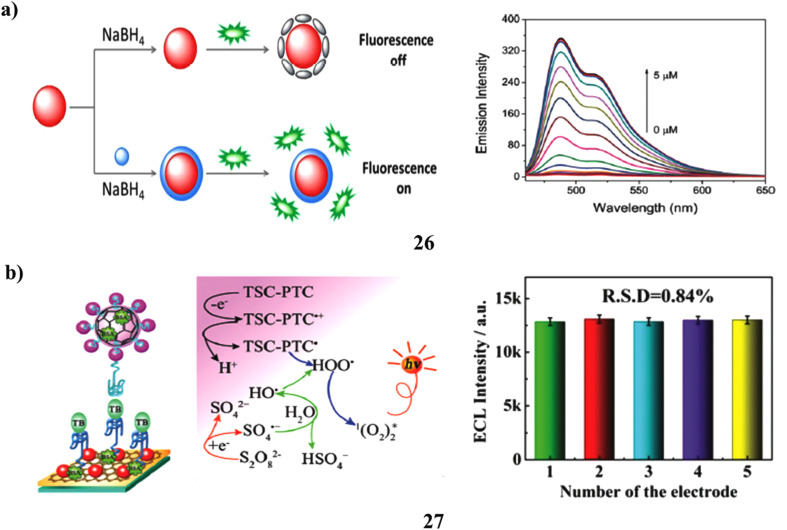
(a) Fluorescence recovery assay for Hg^2+^ detection using perylene-AuNP nanoprobes (26) reproduced from ref. 45 with permission from Royal Society of Chemistry Publisher, Copyright 2016. (b) Electrochemiluminescence study of PTCA-TSC-C60-AuNP composite for thrombin sensing (27) reproduced from ref. 46 with permission from Royal Society of Chemistry Publisher, Copyright 2015.

A new fluorescence turn-on technique for Hg^2+^ ion detection has been developed using Au nanoparticles and a perylene probe (26). It was shown that strong hydrophobic and electrostatic interactions allowed the perylene probe to adsorb onto the Au-NP surface. The inclusion of Au nanoparticles considerably reduced the probe's fluorescence. The surface of the Au nanoparticles formed an Au/Hg amalgam as a result of the reduction of Hg^2+^ following the addition of NaBH_4_. Consequently, the perylene probe exhibited minimal adsorption and quenching by the Au/Hg amalgam. Thus, it was possible to detect a turn-on fluorescence signal. The assay demonstrates considerable sensitivity, with the capability to detect Hg^2+^ concentrations as low as 5 nM. Its exceptional selectivity is further demonstrated by the evaluation of several metal ions, which revealed no discernible interference. The assay has also been effectively applied for the quantification of Hg^2+^ in lake water samples. Consequently, a simple, quick, economical, highly sensitive, and selective Hg^2+^ sensing method has been developed (Fig. 15a).^45^ A novel AuNPs/TSC-PTC/C_60_ nanocomposites-based electrochemiluminescent (ECL) signal tag (27) was created for the detection of thrombin (TB). C_60_ nanoparticles were functionalized with PTCA and TSC to create AuNPs, which allowed for aptamer (TBA 2) tagging for signal amplification in the nanocomposite. The S_2_O_8_^2−^/O_2_ system showed a much improved ECL response when the modified glassy carbon electrode (GCE) containing AuNPs/graphene and thiolated TBA 1 formed a sandwich-type structure with TB and TBA 2. The ultralow detection limit of 3.3 fM and broad linear range (1 × 10^−5^–10 nM) demonstrated the aptasensor's exceptional sensitivity (Fig. 15b).^46^

**Fig. 16:**
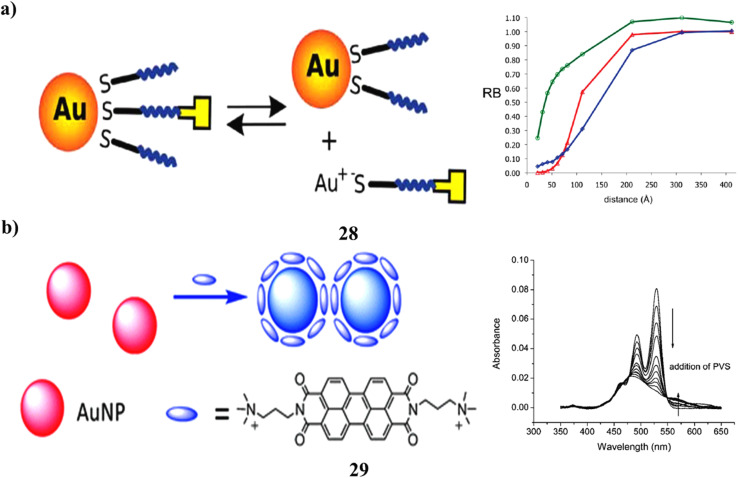
(a) Fluorescence quenching of perylene-monoimide-disulphide-AuNP hybrids (28) reproduced from ref. 47 with permission from Royal Society of Chemistry Publisher, Copyright 2010. (b) UV-vis spectra showing aggregation-induced color change in perylene probe-AuNPs (29) reproduced from ref. 48 with permission from Royal Society of Chemistry Publisher, Copyright 2011.

The interaction between disulfides and gold nanoparticles was investigated using fluorescence spectroscopy with a perylene-monoimide dye linked to a dissymmetric disulfide through a tetraethylene glycol alkyl chain (PMImSS). Quantum chemical calculations employing the polarizable continuum model (PCM) predicted strong quenching of perylene-monoimide fluorescence by AuNPs, attributed to efficient excitation energy transfer from the dye to the nanoparticle. This prediction was experimentally confirmed, as fluorescence quenching occurred upon introducing unmodified AuNPs into a PMImSS solution. Fluorimetric titration curves suggested an equilibrium between free and bound ligands, despite the generally higher affinity of thiols and disulfides for gold surfaces. Fully functionalized PMImSS-AuNP hybrids (28) were then synthesized and purified. However, fluorescence correlation spectroscopy revealed the persistence of free PMImSS ligands in dilute (pM-level) suspensions of these functionalized nanoparticles over several days (Fig. 16a).^47^ The aggregation of Au-NPs was induced by a cationic perylene probe (29). The probe-free monomer possesses a substantial planar aromatic ring structure that can be efficiently adsorbed onto the surface of the gold nanoparticles. Rapid Au-NP aggregation, visible UV-vis spectra, and color changes in the solution were caused by the strong π–π stacking and hydrophobic interactions between probe molecules adsorbed on neighboring nanoparticles, as well as the neutralization of the citrate ion negative charges on the Au-NP surface. It has been shown that this finding may be used for label-free selective sensing of mercury ions (Fig. 16b).^48^

**Fig. 17:**
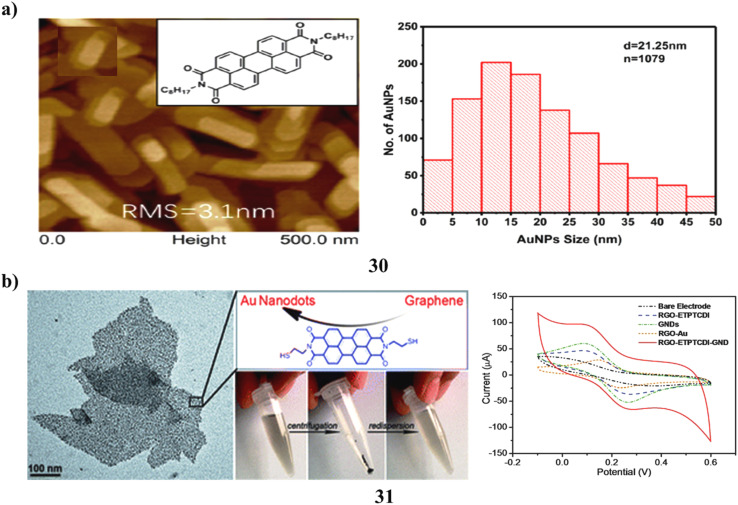
(a) Enhanced photodetector performance of PTCDI-C_8_ organic phototransistor with AuNPs (30) reproduced from ref. 49 with permission from Elsevier Publisher, Copyright 2021. (b) Electrochemical analysis of graphene-perylene-thiol–gold nanodot hybrids (31) reproduced from ref. 50 with permission from Royal Society of Chemistry Publisher, Copyright 2011.

Metal nanostructures demonstrate unique physical characteristics that differ significantly from those of macroscopic objects, and they have found extensive applications in organic optoelectronic devices. *N*,*N*′-Dioctyl-3,4,9,10-perylene tetracarboxylic diimide (PTCDI-C8) is recognized as one of the few air-stable, high-mobility n-type semiconductors, commonly utilized for the fabrication of green or white light detectors due to its principal absorption concentrated within the wavelength range of 450–600 nm. A high-performance red light sensor was realized through an organic phototransistor (OPT) based on PTCDI-C8, enhanced by an optimized layer of gold nanoparticles (AuNPs) (30). The photosensitivity, photoresponsivity, specific detectivity, and external quantum efficiency (EQE) achieved were measured at 6.21 × 10^5^, 24.12 A W^−1^, 2.85 × 10^11^ Jones, and 453.58% at a wavelength of 660 nm, respectively. Furthermore, the AuNPs-modified OPT exhibited a marked enhancement in EQE under green light illumination at 532 nm compared to the OPT constructed solely with the PTCDI-C8 layer. The photophysical mechanisms underpinning the incorporation of AuNPs to enhance device performance were thoroughly investigated (Fig. 17a).^49^ Graphene sheets decorated with metal nanoparticles are emerging as advanced graphene-based hybrids that integrate the unique properties of both components for research and technological applications. A novel wet-chemistry strategy has been established to uniformly decorate reduced graphene oxide (RGO) with gold nanodots (GNDs). This process relies on the non-covalent self-assembly of a perylenethiol derivative (31, ETPTCDI) on the basal plane of graphene oxide (GO), which simultaneously promotes GO reduction and provides a two-dimensional template for the *in situ* nucleation and growth of gold nanodots through thiol–Au interactions. Detailed characterization confirmed the uniform distribution of \∼2 nm GNDs across the RGO-ETPTCDI sheets, enabled by the ordered assembly of ETPTCDI molecules. The resulting RGO-ETPTCDI-GND hybrid exhibits excellent dispersibility, stability, and processability. The uniformly anchored nanodots enhance contrast, enabling clearer visualization of graphene's fine surface structure. Furthermore, electrochemical studies using RGO-ETPTCDI-GND modified glassy carbon electrodes demonstrated markedly enhanced activity. These findings highlight RGO-ETPTCDI-GND as a robust and multifunctional hybrid electrode material with strong potential in electrochemical sensing and energy conversion technologies (Fig. 17b).^50^

**Fig. 18:**
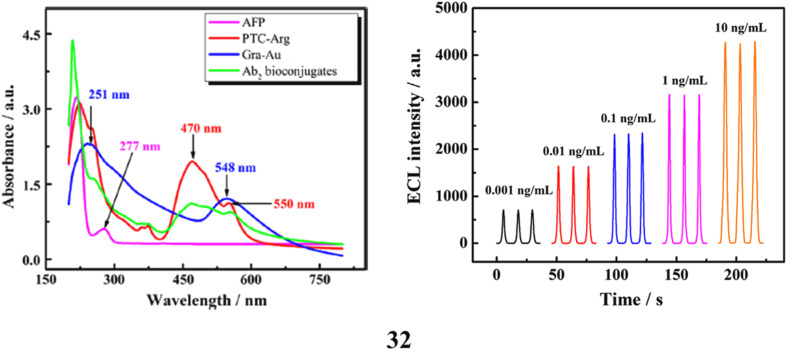
Electrochemiluminescent immunosensor of PTCA-graphene-AuNP hybrid for AFP biomarker detection (32) reproduced from ref. 51 with permission from Elsevier Publisher, Copyright 2013.

A novel electrochemiluminescent immunosensor (32) was developed for the detection of cancer biomarkers based on peroxydisulfate cathodic electrochemiluminescence, utilizing an innovative enhancement of a specific class of perylene derivatives synthesized through the covalent integration of arginine onto 3,4,9,10-perylenetetracarboxylic acid (PTCA). Through supramolecular assembly, the aromatic compound PTC-Arg was successfully immobilized onto gold nanoparticle-functionalized graphene (Au@Gra) *via* π–π stacking, forming PTC-Arg/Au@Gra hybrids. These complexes were employed as multifunctional nanocarriers for secondary antibody (Ab_2_) adsorption, enabling a sandwich-type immunoassay through binding with alpha-fetoprotein (AFP) and the primary antibody (Ab_1_). When applied as an enhancer in the peroxydisulfate system, the PTC-Arg/Au@Gra-based immunosensor exhibited a wide dynamic range of 0.001–10 ng mL^−1^ and an impressive detection limit of 0.3 pg mL^−1^. This indicates substantial potential for development and applications in sensitive bioassays for clinical detection (Fig. 18).^51^

Perylene-based gold nanoparticles (Au-NPs) integrate the exceptional photostability and strong fluorescence of perylene dyes with the surface plasmonic properties of gold nanoparticles, forming robust nanomaterials suitable for optical sensing, bioimaging, and electronic applications. The principle behind these systems lies in the π–π stacking ability and photoactive nature of perylene derivatives, which, when conjugated to Au-NPs, can participate in energy transfer processes such as fluorescence quenching or enhancement depending on the distance and orientation of the dye relative to the gold surface. In terms of performance, perylene-functionalized Au-NPs exhibit excellent photostability, high fluorescence quantum yield, and strong absorption/emission in the visible spectrum, making them ideal for long-term imaging and environmental monitoring. The detection mechanism typically involves fluorescence-based or plasmon-enhanced sensing, where interaction with target analytes alters the electronic environment of the perylene moieties, leading to detectable optical changes. These systems offer high selectivity when perylene is functionalized with receptor units that can recognize specific ions, biomolecules, or pH changes. Advantages include superior optical stability, strong signal intensity, and resistance to photobleaching, as well as the ability to form ordered nanostructures due to the planar geometry of perylene. On the other hand, disadvantages include potential aggregation due to strong π–π interactions, limited solubility in aqueous media, and possible fluorescence quenching at high surface loading. Nevertheless, Au-NPs based on perylene are a promising class of photoactive nanomaterials with a wide range of uses in imaging, sensing, and nanophotonic devices. Collectively, perylene-functionalized AuNPs offer superior photostability, high fluorescence quantum efficiency, and excellent resistance to photobleaching, distinguishing them from fluorescein- and pyrene-based systems. Their quantitative performance metrics include enhanced photoresponsivity (24.12 A W^−1^) and EQE up to 453% in AuNP-integrated phototransistors, highlighting their suitability for optoelectronic devices and long-term sensing. Nevertheless, π–π stacking between perylene units often leads to aggregation and limited solubility in aqueous media, which restricts biological applications. Consequently, these systems are particularly promising for stable, solid-state optoelectronic, sensing, and energy conversion applications requiring high optical robustness.

## 5. Pyrene-based gold nanoparticles

**Fig. 19:**
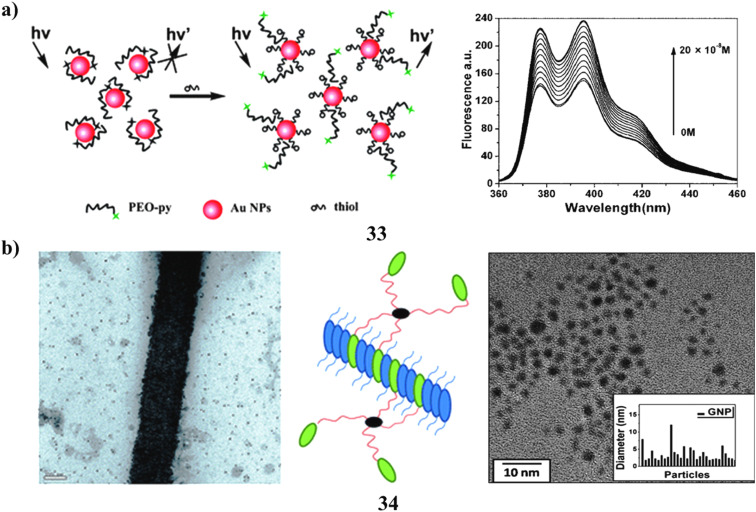
(a) Fluorescence emission of PEGylated pyrene-AuNPs for biothiol detection (33) reproduced from ref. 52 with permission from Royal Society of Chemistry Publisher, Copyright 2010. (b) TEM morphology showing π–π stacking of pyrene-thiol-functionalized AuNPs (34) reproduced from ref. 53 with permission from Royal Society of Chemistry Publisher, Copyright 2015.

A highly soluble fluorescent pyrene derivative exhibiting significantly enhanced fluorescence intensity in aqueous buffer was synthesized utilizing a PEGylation approach. The very effective energy transfer between PEO-Py and Au NPs allowed the highly soluble PEGylated pyrene (PEO-Py) to be non-covalently adsorbed onto their surface, forming dyads with quenched fluorescence. The sensitive turn-on fluorescence detection of biothiols was carried out using the PEO-Py/Au NPs dyads (33) as the detector. The restoration of PEO-Py's fluorescence following the addition of cysteine (Cys) suggests that Cys can efficiently alter the kinetics of energy transfer between PEO-Py and Au NPs. This phenomenon made it easier to detect Cys with a limit of detection (LOD) of 11.4 nM. A linear range of 1.25 × 10^−8^ to 2.25 × 10^−7^ M was developed for the determination of Cys. The detection method was notably unaffected by any of the other amino acids present in proteins. It is important to note that the PEO-Py/Au NPs system's detection sensitivity was more than five times higher than that of the Py/Au NPs system. This sensor device was also able to detect glutathione and other biothiol compounds. The process for determining the overall amount of aminothiols in human plasma was carried out effectively. The result is a fluorescent probe that is easily made, inexpensive, and highly soluble, and its effectiveness in identifying a range of biological analytes of interest is anticipated (Fig. 19a).^52^ Gold nanoparticles functionalized with 11-(4-(pyren-1-yl)phenoxy)undecane-1-thiol (34) were synthesized and characterized. The TEM imaging of these nanoparticles revealed comparable particle sizes, approximately 2.5 nm, substantiated by the absorption spectra. Through the co-self-assembly of the pyrene derivative in conjunction with these functionalized nanoparticles, varying morphologies of Au nanoparticles were observed *via* TEM. In comparison to Au nanoparticles functionalized with 1-dodecanethiol, pyrene-thiol-capped gold nanoparticles exhibited superior distribution on the self-assembled nanostructures of the pyrene derivative, facilitating π–π interactions. This outcome reflects the engineered manipulation of nanoparticles through diverse functionalities, which could signify a notable advancement in the realm of small-scale device applications (Fig. 19b).^53^

**Fig. 20:**
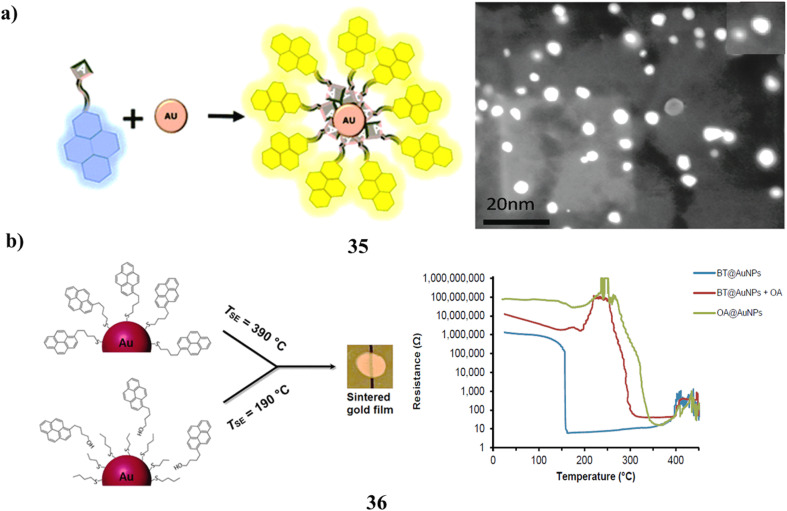
(a) Excimer emission behavior of pyrene-adenine-AuNPs (35) reproduced from ref. 54 with permission from Royal Society of Chemistry Publisher, Copyright 2021. (b) Thermal stability of pyrene-butanethiol-AuNPs under sintering conditions (36) reproduced from ref. 55 with permission from American Chemical Society Publisher, Copyright 2017.

The functionalization of gold nanoparticles with a pyrene fluorophore (35) *via* a nucleobase spacer serves as a connecting moiety. The resultant gold nanoparticles exhibit intrinsic photoluminescence characteristics and are not subject to quenching by the surface plasmons of gold nanoparticles. The emergence of excimer emission corresponding to pyrene further substantiates the proposed arrangement of the pyrene entities on the gold surface in a densely packed configuration (Fig. 20a).^54^ Understanding the sintering behavior of gold nanoparticles (AuNPs) is crucial for developing advanced materials in printed electronics, catalysis, and sensing. This study evaluates the ability of different compounds to stabilize AuNPs (36) against thermal sintering, comparing those with surface-anchoring functional groups to those without. Thermal stability was examined using thermogravimetric analysis, scanning electron microscopy, and the temperature of the sintering event (TSE). Results show that strongly anchored stabilizers provide superior protection, preventing nanoparticle sintering until their thermal decomposition occurs. These findings highlight the importance of molecular anchoring in enhancing the thermal durability of AuNP-based nanomaterials for practical applications. As a stabilizer, 1-pyrenebutanethiol was used to get a TSE of 390 °C. Two unanchored stabilizers were discovered to be very successful when paired with butanethiol-capped AuNPs: oleylamine (TSE ≈ 300 °C) and a derivative of perylenedicarboximide (TSE ≈ 540 °C). The latter gave ligand-stabilized AuNPs an unparalleled degree of thermal stability. The results show that in order to guarantee a homogeneous combination of AuNPs and stabilizer inside a film, it is crucial to pick stabilizers lacking anchoring groups that have an affinity for the capping ligands on the AuNPs (Fig. 20b).^55^

**Fig. 21:**
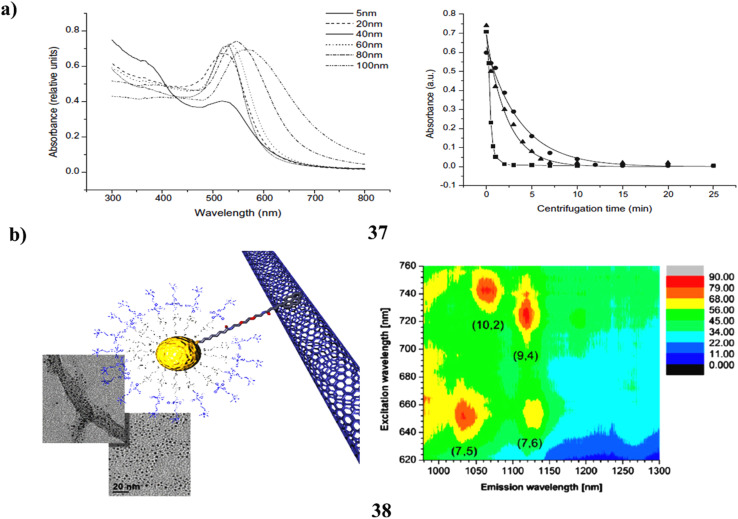
(a) UV-vis absorption spectra for benzo[*a*]pyrene extraction using AuNPs (37) reproduced from ref. 56 with permission from Elsevier Publisher, Copyright 2010. (b) TEM image of pyrene-SWCNT-AuNP hybrid nanostructures showing noncovalent interaction (38) reproduced from ref. 57 with permission from American Chemical Society Publisher, Copyright 2014.

The pronounced affinity observed between polycyclic aromatic hydrocarbons (PAH) and the surface of gold colloids (37) is systematically examined to develop a method for the extraction of water samples. The best extraction efficiencies for all analytes examined were shown by the 20 nm gold nanoparticles within the 20–100 nm particle diameter range. This novel methodology is integrated with laser-excited time-resolved Shpol'skii spectrometry for the direct assessment of benzo[*a*]pyrene in drinking water samples. For a sample volume of 500 µL, the analytical metrics indicate a precise and accurate analysis at the parts-per-trillion concentration level. The extraction efficiencies are statistically comparable to 100%, with relative standard deviations remaining below 2%. The average recovery rates fluctuated between 87.5% and 96.5% for varying analyte concentrations. The straightforward nature of the experimental procedure, combined with low analytical costs and exemplary analytical metrics, underscores the potential of this methodology for the routine analysis of drinking water samples (Fig. 21a).^56^ The Synergistic effects and improved functionality are expected when several nanostructures are combined to create a multifunctional hybrid. Carbon nanotubes and gold colloids are well-studied building blocks with unique optical and electrical characteristics that may be used into a range of optoelectronic applications. This framework presents a novel approach to the manufacture of water-soluble nanohybrids *via* noncovalent interactions between AuNPs and SWCNTs co-functionalized with choline and pyrenyl (38) residues. A procedural methodology employing consecutive centrifugation steps facilitates the straightforward isolation of the conjugates in their water-soluble form. To characterise the resulting nanohybrids and confirm that the only mechanism causing the formation of the water-soluble nanohybrids is the interaction of the pyrene residues on the co-functionalized AuNPs with the nanotube walls, each step of the process was methodically observed using TEM and UV spectroscopy. The contact between the AuNPs and the SWCNTs is apparently noncovalent, as shown by further analyses such as Raman spectroscopy and photoluminescence mapping. This demonstrates that the near-infrared emission is successfully preserved by the nanotubes. Studies of carbon nanotubes suppressing pyrene's fluorescence provide light on their interactions. The determination of association constants is made possible by the observed quenching behaviour. The strength of the complex formation between functionalised AuNPs and SWCNTs is shown by these values. The findings establish a foundation for innovative strategies for the synthesis of water-soluble nanohybrids between AuNPs and SWCNTs rooted in noncovalent interactions (Fig. 21b).^57^

**Fig. 22:**
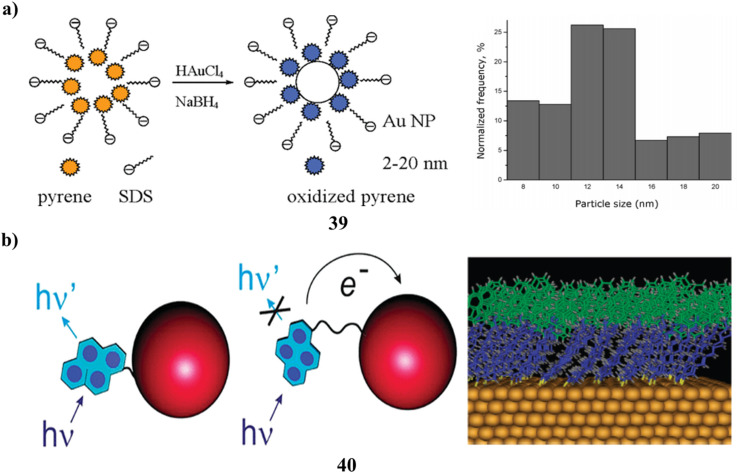
(a) MEKC analysis of pyrene-SDS-AuNP systems showing controlled particle formation (39) reproduced from ref. 58 with permission from American Chemical Society Publisher, Copyright 2005. (b) Computational modelling of fluorescence properties in pyrene-linked AuNPs (40) reproduced from ref. 59 with permission from American Chemical Society Publisher, Copyright 2008.

Gold nanoparticles synthesized *via* chemical reduction in sodium dodecyl sulfate (SDS) solution exhibit size regulation through the incorporation of pyrene. Micellar electrokinetic capillary chromatography (MEKC) is employed to analyze the dimensions and polydispersity of gold nanoparticles, demonstrating that pyrene significantly influences the reduction of size and the tightening of dispersity. The MEKC electropherograms also indicate that pyrene may undergo oxidation by aqueous Au(iii) complexes. Subsequently, all reduced Au complexes (39) were solubilized within the pyrene-SDS micelles. The proliferation of gold nanoparticles beyond the initial formation stage was effectively curtailed by the enclosing SDS and electrophilic pyrene (Fig. 22a).^58^ Gold nanoparticles modified with chromophores (40) are recognized for displaying unpredictable fluorescence correlating with their structural characteristics. Odd–even effects, attributable to the number of methylene units in the chain linking to the fluorophore, as well as the characteristics of the anchoring group on the gold surface, have previously been implicated in this behavior. The fluorescence phenomena of two newly synthesized pyrene derivatives tethered to gold nanoparticles are investigated. Two ligands, structurally identical yet differing solely in their anchoring groups, were synthesized and conjugated to the gold nanoparticles. Identical alterations in fluorescence characteristics, specifically a red spectral shift accompanied by a moderate enhancement of quantum yield and a reduction in excited-state lifetime, were observed in both instances and are attributed to the proximity of the gold core. By contrasting these findings with those reported for alternative pyrene derivatives, it becomes evident that the nature of the binding group does not influence the fluorescence properties of the fluorophores attached to the nanoparticle surface. The most intense fluorescence occurs when pyrene is separated from the gold by a brief alkyl chain. This atypical behavior can be elucidated through the interplay of competing chain–chain and chromophore–chromophore interactions, utilizing appropriate energy diagrams (Fig. 22b).^59^

Pyrene-based Au-NPs are photoactive nanomaterials that exploit the strong fluorescence, π–π stacking interactions, and environment-sensitive emission properties of pyrene to develop responsive systems for sensing, imaging, and molecular recognition. The principle centers on the conjugation of pyrene moieties to the surface of Au-NPs, where their photophysical behavior is influenced by proximity to the gold core, leading to fluorescence quenching or modulation through mechanisms like FRET. In terms of performance, these systems demonstrate high sensitivity and tunable emission, particularly due to pyrene's ability to form excimers, which provide distinct emission profiles useful for ratiometric sensing. The detection mechanism typically relies on fluorescence changes in response to environmental factors, which can alter the monomer-to-excimer emission ratio. Pyrene-functionalized Au-NPs exhibit strong selectivity when modified with specific receptor groups or recognition units, enabling selective detection of metal ions, aromatic pollutants, or biomolecules. Advantages include high fluorescence intensity, excellent photostability, and the ability to monitor molecular interactions in real-time. However, disadvantages involve limited aqueous solubility of pyrene derivatives, potential aggregation due to strong π–π interactions, and fluorescence quenching at high surface density or upon nanoparticle clustering. Despite these challenges, pyrene-based Au-NPs offer a versatile platform for designing advanced optical probes and responsive nanodevices in chemical and biomedical applications. Pyrene-modified AuNPs exhibit distinctive dual fluorescence behaviour arising from monomer and excimer emissions, providing ratiometric sensitivity to environmental polarity and molecular interactions. Their quantitative performance includes detection limits down to 11.4 nM for cysteine and thermal sintering resistance up to 390 °C, underscoring their versatility in both sensing and materials applications. Compared with perylene-based systems, pyrene-AuNPs afford broader emission tunability but are limited by aggregation and poor aqueous solubility. These nanohybrids are best suited for chemical sensing, pollutant monitoring, and nanophotonic device fabrication where excimer-based ratiometric response and fluorescence stability are required.

A summary of molecules, principles, detection mechanisms, and key challenge is found in Table 1 below.

**Table 1:** A structured overview of the photoresponsive-based gold nanoparticles (Au-NPs), including their principle, performance, detection mechanism, quantitative metrics, key challenges and applications, is provided in this table

Molecule	Principle	Performance	Detection mechanism	Applications	Quantitative metrics	Key challenges/limitations
Azobenzene	Reversible *trans*–*cis* photoisomerization under UV/visible light alters surface polarity and aggregation	Rapid (40–120 s) and reversible switching; tunable optical response	Plasmonic shift or FRET modulation during light-induced switching	Light-driven actuators, drug release, and optical switches	Photothermal efficiency ≈ 35%; relaxation time 82–125 min	UV activation limits tissue penetration; photofatigue after multiple cycles
Fluorescein	Fluorescence modulation by proximity to AuNP surface and environmental pH	High quantum yield (≈0.9); bright, tunable emission	FRET- or fluorescence-based sensing	Bioimaging, immunosensing, and environmental assays	LOD (OTA): 0.02 ng mL^−1^; LOD (Cu^2+^): 0.37 nM	Photobleaching; fluorescence instability under variable pH
Perylene	π–π stacking interactions and plasmon-coupled fluorescence	Excellent photostability and emission intensity; strong visible absorption	Fluorescence quenching/enhancement by analyte binding	Optoelectronic devices, Hg^2+^ sensing, bioassays	EQE: 453%; photoresponsivity: 24.12 A W^−1^	Aggregation and poor water solubility
Pyrene	Monomer–excimer emission and π–π stacking-driven optical modulation	Dual fluorescence with ratiometric sensitivity	Excimer/monomer intensity ratio and FRET effects	Biothiol sensing, pollutant detection, and nanophotonics	LOD (Cys): 11.4 nM; TSE: 390 °C	Aggregation; limited solubility; fluorescence quenching at high loading

## 6. Conclusion

Photoresponsive AuNPs offer powerful opportunities for light-mediated control of biological and material functions. Their tunable optical properties and surface versatility make them valuable in therapeutic, diagnostic, and sensing applications. However, translation remains constrained by limited light penetration, long-term biocompatibility, and fabrication reproducibility. Future progress lies in the development of NIR-activated, multi-stimuli, and biodegradable systems, supported by AI-assisted molecular design and scalable synthesis. With these advances, photoresponsive AuNPs are poised to evolve from experimental nanostructures to clinically relevant smart materials for precision healthcare.

## Abbreviations

Au-NPs: Gold Nanoparticles
SPR: Surface Plasmon Resonance
NIR: Near-Infrared
PDA: Polydopamine
DSC: Differential Scanning Calorimetry
UV-vis: Ultraviolet-Visible
AFM: Atomic Force Microscopy
TEM: Transmission Electron Microscopy
AB: Azobenzene
RMD: Reactive Molecular Dynamics
SERS: Surface Enhanced Raman Spectroscopy
OTA: Ochratoxin A
AMF: Amino-Methyl Fluorescein
FRET: Fluorescence Resonance Energy Transfer
ROS: Reactive Oxygen Species
EGFR: Epidermal Growth Factor Receptor
FITC: Fluorescein Isothiocyanate
CLSM: Confocal Laser Scanning Microscope
MEF: Metal-Enhanced Fluorescence
Cys: Cysteine
HCys: Homocysteine
GSH: Glutathione
NSET: Nanometal Surface Energy Transfer
NHCs: N-Heterocyclic Carbenes
PDI: PeryleneDiimide
ECL: Electrochemiluminescent
TSC: Thiosemicarbazide
PTCA: 3,4,9,10-Perylene Tetracarboxylic Acid
OPT: Organic Phototransistor
PCM: Polarizable Continuum Model
EQE: External Quantum Efficiency
RGO: Reduced Graphene Oxide
GNDs: Gold Nanodots
AFP: Alpha-Fetoprotein
LODs: Detection of Limits
PAH: Polycyclic Aromatic Hydrocarbons
SWCNTs: Single-Walled Carbon Nanotubes
MEKC: MicellarElectrokinetic Capillary Chromatography
PET: Photoinduced Electron Transfer

## Conflicts of interest

There are no conflicts to declare.

## Data Availability

No primary research results and no new data were generated or analysed as part of this review.
